# Lifestyle-attributable burden of young-onset stroke in Chinese and global populations aged 20–54 years: A three decades comparative study (1990–2021) using Global Burden of Disease study data

**DOI:** 10.18332/tid/208007

**Published:** 2025-08-29

**Authors:** Danrui Yang, Guohan Fan, Jiajia Cheng, Qingzhou Meng, Keyang Liu, Kokoro Shirai, Hiroshi Yatsuya, Ren Chen, Yan Zhang

**Affiliations:** 1 School of Health Service Management, Anhui Medical University, Hefei, China; 2 Graduate School of Medicine, Osaka University, Osaka, Japan; 3 Graduate School of Medicine, Nagoya University, Nagoya, Japan; 4 Department of Geriatrics, Chaohu Hospital of Anhui Medical University, Hefei, China

**Keywords:** mortality, lifestyle risk factors, disability-adjusted life years, young-onset stroke, China-global comparison

## Abstract

**INTRODUCTION:**

Young-onset stroke represents a growing public health crisis globally along with rapid lifestyle changes. This study investigated the mortality and disability burden of young-onset stroke attributable to modifiable lifestyle factors in China compared with global trends, aiming to identify critical intervention targets.

**METHODS:**

Utilizing Global Burden of Disease 1990–2021 data, we analyzed age-stratified mortality and disability-adjusted life years for four lifestyle risk factors (tobacco, high alcohol use, dietary risk, and low physical activity). Temporal trends were assessed through joinpoint regression and age-period-cohort modeling, with population-attributable fractions compared between Chinese and global populations over three decades.

**RESULTS:**

China demonstrated persistently greater burdens from tobacco and high alcohol use than global averages did, particularly among males, where alcohol-attributable disability-adjusted life years (DALYs) increased 21% faster than mortality rates did. Gender disparities were prominent, with male population attributable fractions (PAFs) for alcohol exceeding female levels by 9.3-fold. Paradoxically, Chinese females presented rising low physical activity-related DALYs despite declining mortality. Midlife adults (aged ≥40 years) showed accelerated risk accumulation, whereas dietary risk reductions in China outpaced global declines by 2.4-fold. Age effects for all risks were weaker than global estimates, although period and cohort patterns aligned closely.

**CONCLUSIONS:**

The diverging trajectories of mortality and disability burdens underscore China’s dual challenge: curbing substance-related mortality in young males while containing midlife disability escalation. Culturally tailored strategies addressing gender-specific risk profiles and alcohol-DALY decoupling are urgently needed. These findings provide pivotal evidence for global stroke prevention in transitioning societies.

## INTRODUCTION

Stroke ranks as the second leading cause of death and the third leading contributor to the combined burden of mortality and disability worldwide^1^. Traditionally, stroke has been viewed as predominantly affecting older adults. However, recent studies indicate a worrying trend of young-onset stroke, defined as stroke occurring in younger individuals (e.g. under 55 years of age)^2^, with the age of stroke onset shifting toward younger populations^3,4^. This growing burden of young-onset stroke underscores the need for targeted prevention and management strategies in younger populations.

Studies have shown that modifiable risk factors (such as tobacco use and high alcohol use) may play pivotal roles in stroke onset and progression. Promoting healthy lifestyle changes through public health interventions can significantly reduce the risk of serious health issues, including stroke^5^. However, this association may be mediated through a complex interplay of sociocultural contexts, socioeconomic disparities, and environmental determinants^6^. Ecological analyses have revealed significant cross-national variations in stroke burden due to lifestyle factors, with notable contributions in low- and middle-income countries, possibly influenced by macro-level factors such as healthcare infrastructure and national development^7^.

Stroke has been the leading cause of disability and mortality among adults in China since 2015 ^8^. In China, the stroke burden remains focused on older adults but now shows a growing pattern in younger age groups^9^. The population at risk for stroke is becoming younger, with individuals aged 40–64 years accounting for approximately three-quarters of those identified as high risk^10^. A recent study revealed that 62% of young Chinese individuals are physically inactive, with smoking and alcohol consumption rates of 20.8% and 15.2%, respectively, indicating a potential future increase in stroke burden^11^. Although extensive research has established the association between unhealthy lifestyle factors and stroke risk, the burden of young-onset stroke attributable to these highly prevalent risk factors remains poorly quantified. This knowledge gap warrants further epidemiological investigations to better understand the age-specific impact of lifestyle factors, which is crucial for developing targeted preventive strategies tailored to younger adults at risk.

Therefore, this study aims to: 1) examine the contributions of four prevalent lifestyle factors (tobacco, high alcohol use, poor diet, and low physical activity) to stroke burden (mortality and disability) among young and middle-aged adults in China; and 2) compare these trends with global levels from 1990 to 2021. This study seeks to identify China’s unique challenges in addressing young-onset stroke and contributes to global efforts to reduce the impact of stroke by informing national prevention strategies.

## METHODS

### Data sources and study design

Stroke mortality and disability-adjusted life years (DALYs) data, and associated risk factor estimates, were extracted from the Global Burden of Disease Study 2021 (GBD 2021), with a focus on China-specific analyses at the national level. As the most comprehensive epidemiological modelling platform to date, GBD 2021 systematically quantifies health loss from 371 diseases/injuries and 88 risk factors across 204 nations and territories via a standardized methodology. The data sources of GBD studies primarily include population censuses, household surveys, vital statistics, disease registries, Centers for Disease Control and Prevention reports, and international websites, among others^12^.

Specifically, age-stratified (20–54 years) estimates of stroke mortality, DALYs, and risk factor exposure were extracted for both Chinese and global reference populations (1990–2021). In alignment with established protocols of GBD 2021^12^, this analysis examined four key modifiable lifestyles: 1) tobacco use, incorporating data on active smoking and chewing tobacco as well as secondhand smoke exposure; 2) high alcohol use, referring to alcohol consumption in excess of the theoretical minimum risk exposure level; 3) dietary risks, including both protective factors (e.g. fruit, vegetable, and whole grain consumption) and risk-elevating components (e.g. sodium intake, processed meat consumption); and 4) low physical activity, assessed via standardized metabolic equivalent (MET) thresholds on the basis of internationally established criteria for energy expenditure assessment. These indicators were developed through GBD’s hierarchical measurement framework, which integrates diverse data sources while maintaining cross-country comparability.

### Ethics

This study used publicly available, aggregated, and anonymized data from the GBD Study, which comply with the ethical standards of the original data providers. No direct human participants were involved, and therefore no additional institutional review board (IRB) approval was needed.

### Identification of stroke

Stroke cases were identified according to the criteria of the World Health Organization (WHO) and were defined as rapidly developing focal neurological deficits persisting over 24 hours or leading to death, with neuroimaging-confirmed vascular etiology excluding other potential causes. Consistent with GBD case definitions, stroke cases were classified into three etiological subtypes, namely, ischemic stroke (ICD-10 code I63), intracerebral hemorrhagic stroke (I61) and subarachnoid hemorrhage stroke (I60), as validated through the standardized case verification algorithms of GBD^13^.

### Statistical analysis

Temporal trends in stroke mortality and DALYs attributable to the four modifiable risk factors were analyzed via Joinpoint regression via the Joinpoint regression program (version 5.1.0, National Cancer Institutes). This method, which assumes that the dependent variable counts follow a Poisson distribution (and variance)^14^, offers the advantage of detecting significant changes in trend slopes over time by identifying inflection points (joinpoints). Compared with traditional linear regression, Joinpoint regression provides a flexible, data-driven approach that segments long-term trends into meaningful phases, enabling more precise characterization of temporal patterns in epidemiological measures^15^. The model identified inflection points through Monte Carlo permutation tests (4499 permutations, p<0.05), calculating annual percentage changes (APCs) for each segment via weighted least squares regression. Average annual percent changes (AAPCs) with 95% confidence intervals (CIs) were computed to summarize overall trends across the study period (1990–2021)^14,16^. The population attributable fraction (PAF) quantifies the proportion of disease burden (mortality or disability) attributable to a specific risk factor. It is calculated as:

PAF = (Burden attributable to exposure)/(Total burden in population)×100%

To reflect absolute changes over time, we also calculated the differences in mortality rates and DALY rates between 1990 and 2021 via direct subtraction, complementing the analysis of relative risk changes.

To examine the differences in stroke mortality attributable to lifestyle factors across age, period, and cohort, the age-period-cohort (APC) model (National Cancer Institute of the United States) was applied^17^. This analysis included individuals aged 25–54 years in China and globally, who were stratified into six 5-year age groups (25–29 to 50–54 years). Temporal trends were assessed across six 5-year intervals from 1992–1996 through 2017-2021. Birth cohort effects were examined through ten consecutive 5-year cohorts, spanning individuals born between 1942–1947 and 1987–1992. This comprehensive approach enabled simultaneous estimation of age-specific risks, temporal trends, and generational variations in lifestyle-related stroke mortality patterns.

Stratified analyses were conducted by stroke subtype (ischemic stroke [ICD-10 I63] and intracerebral hemorrhage [I61]) to examine potential heterogeneity in risk factor associations. The subtype-specific analyses followed the same methodological framework as the primary analysis, with separate Jointpoint regression models fitted for each subtype to identify distinct temporal patterns. APC analyses were also performed separately for each subtype to assess potential variations in demographic determinants across different stroke etiologies.

## RESULTS

Table 1 compares the PAFs, temporal trends, and 95% CIs for four modifiable risk factors (tobacco, high alcohol use, dietary risk, and low physical activity) in China and globally from 1990 to 2021. Tobacco use showed higher Chinese PAFs than global averages, with a slower male decline (-0.685; 95% CI: -0.921 – -0.449 vs -2.360; 95% CI: -2.670 – -2.050) annual change in 2021, indicating that although the tobacco-related stroke burden is decreasing in both regions, the reduction is less pronounced in Chinese men. High alcohol use has risen sharply in China (0.288; 95% CI: 0.079–0.498, per year) but has fallen globally (-0.500; 95% CI: -0.675 – -0.324, per year), alongside a 9.2-fold gender gap (males: 0.147 vs females: 0.016, in 2021), reflecting both cultural patterns and gender-specific exposures that may require targeted public health strategies. Dietary risk PAFs were higher in China, though female declines outpaced males (-2.878; 95% CI: -3.343 – -2.410 vs -1.195; 95% CI: -1.429 – -1.960, per year), suggesting some progress in dietary improvements, particularly among women. Low physical activity has accelerated in China (0.455; 95% CI: 0.230–0.280, per year) versus stable global trends, with female PAFs surpassing male PAFs by 2021, indicating that physical inactivity may be an emerging and increasing risk factor for stroke in Chinese women. DALY analyses highlighted divergent trends: tobacco-related DALYs declines lagged globally (-0.908; 95% CI: -1.040 – -0.775) vs -1.477; 95% CI: -1.649 – -1.305, per year); alcohol-attributable DALYs rose 21% faster than mortality in Chinese males (0.483; 95% CI: 0.233–0.733 vs 0.398; 95% CI: 0.237–0.559, per year), indicating a disproportionate increase in disability burden that may reflect long-term health consequences beyond death; and low physical activity drove a 1.6-fold greater increase in DALYs (0.732; 95% CI: 0.614–0.850, per year) than mortality did, disproportionately affecting females (0.217; 95% CI: 0.044–0.390 vs -0.365; 95% CI: -0.682 – -0.047, per year), underscoring a growing impact on stroke-related disability, especially among women.

**Table 1:** Population-attributable fractions (PAFs) of stroke due to tobacco, high alcohol use, dietary risks, and low physical activity among individuals aged 20–54 years in 1990 and 2021, with temporal trends over this period, a secondary analysis of Global Burden of Disease data

*Region*	*Sex*	*Tobacco*			*High alcohol use*			*Dietary risks*			*Low physical activity*		
		*PAF* *1990*	*PAF* *2021*	*AAPC* *(95% CI)*	*PAF* *1990*	*PAF* *2021*	*AAPC* *(95% CI)*	*PAF* *1990*	*PAF* *2021*	*AAPC (95% CI)*	*PAF* *1990*	*PAF* *2021*	*AAPC* *(95% CI)*
**Deaths**													
China	Both	0.452	0.453	-0.983* (-1.147 – -0.819)	0.075	0.112	0.288* (0.079–0.498)	0.314	0.246	-1.748* (-1.899 – -1.598)	0.009	0.013	0.455* (0.230–0.280)
	Male	0.581	0.541	-0.685* (-0.921 – -0.449)	0.113	0.147	0.398* (0.237–0.559)	0.316	0.250	-1.195* (-1.429 – -1.960)	0.006	0.011	1.194* (1.017–1.372)
	Female	0.238	0.214	-2.360* (-2.670 – -2.050)	0.013	0.016	-1.244* (-1.456 – -1.033)	0.312	0.237	-2.878* (-3.343 – -2.410)	0.013	0.021	-0.365* (-0.682 – -0.047)
Global	Both	0.387	0.332	-1.520* (-1.712 – -1.327)	0.060	0.071	-0.500* (-0.675 – -0.324)	0.280	0.235	-1.614* (-1.686 – -1.543)	0.012	0.014	-0.306* (-0.455 – -0.158)
	Male	0.490	0.419	-1.272* (-1.469 – -1.075)	0.087	0.100	-0.336* (-0.582 – -0.090)	0.284	0.237	-1.358* (-1.547 – -1.170)	0.008	0.011	0.017 (-0.092 – 0.125)
	Female	0.233	0.172	-2.441* (-2.586 – -2.296)	0.020	0.018	-1.892* (-2.094 – -1.690)	0.275	0.230	-2.033* (-2.221 – -1.845)	0.017	0.021	-0.616* (-0.616 – -0.785)
**DALYs**													
China	Both	0.447	0.444	-0.908* (-1.040 – -0.775)	0.072	0.107	0.392* (0.141–0.645)	0.316	0.252	-1.600* (-1.720 – -1.481)	0.011	0.018	0.732* (0.614–0.850)
	Male	0.578	0.540	-0.622* (-0.763 – -0.482)	0.110	0.145	0.483* (0.233–0.733)	0.316	0.251	-1.136* (-1.279 – -0.993)	0.007	0.012	1.366* (1.228–1.503)
	Female	0.240	0.215	-2.063* (-2.249 – -1.877)	0.012	0.016	-0.895* (-1.147 – -0.642)	0.316	0.254	-2.442* (-2.762 – -2.121)	0.017	0.031	0.217* (0.044–0.390)
Global	Both	0.383	0.327	-1.477* (-1.649 – -1.305)	0.058	0.068	-0.406* (-0.550 – -0.262)	0.286	0.244	-1.489* (-1.552 – -1.426)	0.015	0.019	-0.100 (-0.208–0.007)
	Male	0.487	0.416	-1.230* (-1.415 – -1.045)	0.084	0.097	-0.250* (-0.385 – -0.114)	0.288	0.244	-1.272* (-1.441 – -1.103)	0.010	0.013	0.159* (0.059–0.259)
	Female	0.236	0.175	-2.293* (-2.364 – -2.222)	0.020	0.0180	-1.617* (-1.793 – -1.440)	0.284	0.246	-1.804* (-1.899 – -1.709)	0.022	0.030	-0.284* (-0.393 – -0.175)

Data based on modeled estimates integrating multiple global sources; exact sample size not applicable. PAF: population-attributable fraction; indicating the proportion of deaths from a certain disease that can be attributed to a certain exposure factor among all deaths from that disease. AAPC: annual average percentage change.

* Indicates that the AAPC value is statistically significant at the α=0.05 level.

Supplementary file Table 1 shows the absolute changes in mortality rates and DALYs rate from 1990 to 2021. During this period, the tobacco-attributable mortality rate among Chinese males declined: -3.539 (95% CI: -6.232 – -0.846) per 100000 population; while Chinese females experienced a reduction: -2.579 (95% CI: -3.522 – -1.636). In contrast, the global smoking-attributable mortality rate decreased: -3.742 (95% CI: -4.804 – -2.680) for males, and -1.976 (95% CI: -2.504 – -1.448) for females. The mortality rate due to dietary risk in Chinese women is higher than the global level, but the absolute change in decline is faster than the global rate (-3.838; 95% CI: -6.842 – -0.834 vs -2.051; 95% CI: -3.054 – -1.048). Compared with mortality, smoking-related DALYs show a greater absolute change. The absolute change in DALYs caused by tobacco among Chinese women exceeds the global average (-117.368; 95% CI: -208.857 – -25.879) vs -96.296; 95% CI: -148.908 – -43.684).

**Figure 1:**
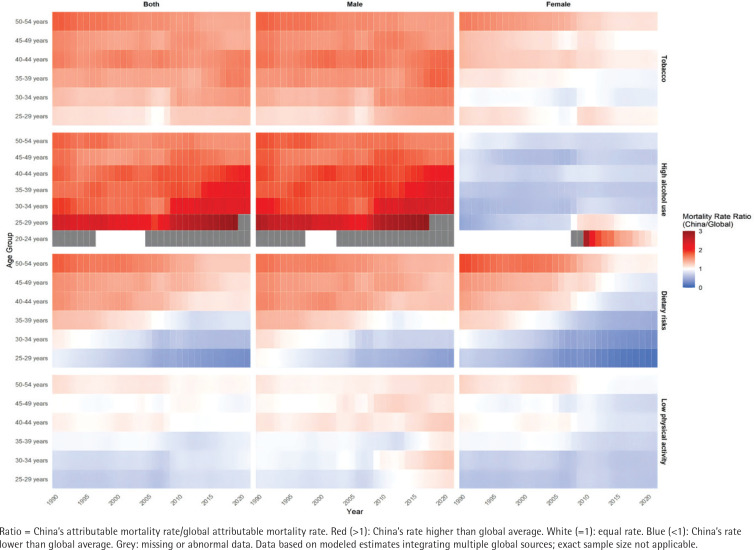
Risk factor-attributable mortality rate ratio for stroke among individuals aged 25–54 years in China versus global average, 1990–2021, a secondary analysis of Global Burden of Disease data

Figure 1 visualizes China’s age-sex specific young-onset stroke mortality rates relative to global averages using a color gradient. The heatmap highlights geographical disparities: tobacco- and alcohol-related mortality dominated red zones (ratio >1), especially in males. Young Chinese males (30–44 years) presented the strongest tobacco-attributable mortality signals, while alcohol-related deaths peaked in males aged 25–44 years. Compared with younger males, older males (45–54 years) presented attenuated red hues, suggesting a reduced risk. Females generally had lower ratios than global averages did, except for a transient cohort aged 20–24 years that has shown elevated risk since approximately 2010. Dietary risks exhibited age-dependent patterns: midlife adults (40–54 years) were red-dominated (high burden), whereas younger adults (25–39 years) presented neutral to protective gradients. Low physical activity mortality rates aligned with global trends in younger populations (<40 years) but diverged significantly in middle-aged and older adults.

Supplementary file Figure 1 shows the ratio of DALY rates by age group and region for young-onset stroke patients attributable to four modifiable risk factors in China compared with global averages. Tobacco-attributable DALYs were higher in most Chinese age groups, especially males. Two female cohorts (25–29 and ≥40 years) exceeded the global ratios. Alcohol-attributable DALYs showed significant sex differences: male ratios consistently surpassed global averages across all ages, with sharp increases in those aged 25–34 years. The female ratios generally remained lower than the global ratios, except for those aged 20–29 years, whose alcohol-DALYs have exceeded worldwide benchmarks since 2010. Dietary risks DALYs aligned with global averages in Chinese adults <40 years but rose sharply in those ≥40 years. Low physical activity DALYs deviated minimally from global trends, although young males (25–34 years) and older adults (≥50 years) presented higher ratios than global averages did over the past decade.

**Figure 2:**
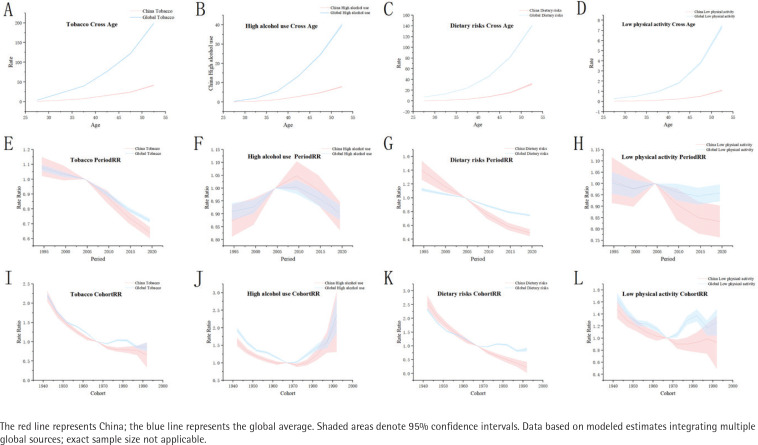
Comparison of age-period-cohort models for stroke mortality rates attributable to lifestyle factors among individuals aged 25–54 years in China and globally, 1992–2021, a secondary analysis of Global Burden of Disease data: Panels (A-D) show age-specific trends attributable to (A) tobacco use, (B) high alcohol use, (C) dietary risks, and (D) low physical activity. Panels (E-H) show period-specific trends attributable to (E) tobacco use, (F) high alcohol use, (G) dietary risks, and (H) low physical activity. Panels (I-L) show cohort-specific trends attributable to (I) tobacco use, (J) high alcohol use, (K) dietary risks, and (L) low physical activity

Figure 2 presents APC analyses of stroke mortality rates and 95% CIs for four modifiable risk factors in China and globally (1992–2021) for ages 25–54 years. Tobacco (Figures 2A, 2E and 2I) global age-specific rates rose sharply (especially for those ≥40 years), whereas trends in the Chinese population declined faster. Cohort risk was higher in earlier Chinese generations. Alcohol (Figures 2B, 2F and 2J) Chinese age trends were stable, contrasting with steeper global increases. Period trends aligned globally, but cohort risk rose sharply in recent generations, indicating generational shifts. Dietary risk (Figures 2C, 2G and 2K) global age trajectories were steeper, with faster periods of decline. Chinese cohorts presented greater historical risk, with a downwards trend in recent generations. Low physical activity (Figures 2D, 2H and 2L) global age-specific rates increased fastest (≥40 years). Period trends declined post-2000, while cohort risks were highest in older Chinese generations.

Supplementary file Figure 2 compares APC analyses of DALYs attributable to tobacco, alcohol, dietary risks, and low physical activity between China and global trends for individuals aged 25–54 years during 1992–2021. For tobacco (Panels A, E and I), global age-specific rates rose sharply after the age of 40 years, whereas period trends in China declined more rapidly. Cohort risk was elevated in earlier generations but markedly decreased in more recent cohorts. With respect to alcohol (Panels B, F and J), the age trends in China were milder than the global patterns. The period effects were similar between China and the global average, but cohort risk increased in recent generations, indicating generational shifts. For dietary risk (Panels C, G and K), the global age trajectories became steeper, while Chinese period trends demonstrated faster declines. Historical Chinese cohorts presented greater risks, with improvements observed in more recent generations. Finally, for low physical activity (Panels D, H and L), global age-related increases were most notable after the age of 40 years. Period trends have declined modestly since 2000, with cohort risk being highest among older Chinese generations.

Additional analyses of early-onset stroke subtypes (Supplementary file Figures 3–10) demonstrated that tobacco use, high alcohol use, and dietary risks conferred higher attributable burdens than global averages did, with pronounced impacts on young males aged 25–29 years, who presented elevated diet-related mortality and alcohol-attributed DALYs. Age effects for these risk factors were consistently lower than global estimates for both mortality and DALYs, whereas period and cohort effects aligned with global trends. For early-onset hemorrhagic stroke, tobacco and alcohol use also exceeded global mortality/DALY burdens; however, young males paradoxically presented lower alcohol-related mortality but higher non-fatal DALYs, suggesting increased morbidity. Adults over 40 years of age had higher dietary risk-associated burdens than the global average. APC decomposition revealed weaker age effects for the studied risks (excluding low physical activity data) than did global estimates, with period/cohort trends mirroring global patterns. Notably, dietary risk rates in China have accelerated beyond global trends.

## DISCUSSION

This comparative study identified three principal divergences in modifiable risk factor burdens for young-onset stroke between China and global estimates. First, China has consistently elevated burdens attributable to tobacco, high alcohol use, and dietary risks relative to the global average, while maintaining low physical activity-attributable burdens comparable to global patterns. Second, significant sex-specific stratification emerged: males presented disproportionate excess burdens for substance-related risks (tobacco/alcohol), whereas females presented distinct epidemiological trajectories marked by concurrent mortality reductions and persistent DALY increases – a paradox notably linked to physical inactivity trends. Third, longitudinal analyses revealed critical temporal dynamics: accelerated midlife risk accumulation beyond the age of 40 years, diverging alcohol-related burden trajectories versus global stability, cohort-dependent risk exposures (historical tobacco/dietary risks versus emerging alcohol patterns in younger generations), and post-2002 mitigation of physical inactivity impacts. These findings collectively underscore the imperative for risk-specific interventions addressing China’s dual challenge of mortality reduction and disability containment within its evolving epidemiological landscape. To date, this study is the first to comprehensively examine both mortality and disability burdens attributable to modifiable lifestyle factors in China while indicating sex-specific and generational disparities that have been underexplored in prior studies.

The disease burden attributable to smoking has significantly decreased since 1990, indicating that tobacco control strategies have achieved certain success. Nevertheless, smoking remains a leading risk factor for disease burden globally, particularly among males^1^. In this study, the high tobacco use-attributable mortality among young Chinese males suggests that tobacco control efforts targeting young males in China remain insufficient. Similarly, China has demonstrated rising alcohol use-attributable mortality rates, in contrast to global declining trends, and the increasing mortality risk among more recent birth cohorts highlights the urgent need for enhanced alcohol control policies targeting younger generations in China. These persistent challenges in tobacco and alcohol control among young Chinese males may be deeply rooted in traditional Chinese social and cultural contexts^18^. Despite the known health risks of tobacco and alcohol, these substances remain integral to social and business interactions in China, especially among young males. The cultural normalization of smoking and drinking is further reinforced by young men’s role as primary economic contributors, increasing their exposure to these risks in occupational settings. Additionally, similar to many countries^19-21^, significantly greater social tolerance to smoking and drinking exists toward young males than toward females. Moreover, previous studies have also indicated that young males tend to exhibit lower health literacy and preventive healthcare awareness^22^, potentially leading to ignorance of smoking and drinking-related health risks. This combination of cultural factors and health behavior patterns may contribute to increased exposure to risk factors and, consequently, elevated early-onset stroke mortality in younger Chinese males. In support of our findings, a large Japanese study showed that modifiable risk factors (e.g. obesity, hypertension) have a stronger impact on cardiovascular disease risk in younger people, underscoring the need for early preventive measures aimed at modifying these controllable risk factors to reduce stroke and related conditions^23^. In addition, recent evidence from a large Korean cohort highlights the significant association between cumulative alcohol burden and increased stroke risk among young adults, emphasizing alcohol consumption as a critical modifiable risk factor in younger populations. These findings reinforce the urgent need to incorporate targeted alcohol reduction strategies within stroke prevention programs for young adults, complementing broader efforts addressing tobacco and other lifestyle risks^24^.

Our findings revealed declining trends in dietary risk-attributable stroke mortality both in China and globally. This improvement might reflect increased dietary awareness and better food accessibility in recent decades worldwide. Consistent with recent evidence from East Asian countries, low consumption of whole grains and legumes and high sodium intake remain leading dietary risk factors contributing to stroke burden, with notable variations between sexes and countries. For example, while Japan and South Korea have experienced declines in diet-related cardiovascular mortality, China has shown increasing trends, particularly among males^25^. However, the emergence of physical inactivity as a growing concern, with faster increasing trends in China compared with global patterns, suggests a transition toward low physical activity and a more sedentary lifestyle^26,27^. Rapid urbanization and economic development in China are likely driving these transitions. While improved living standards have enhanced diet and food security, modern lifestyle changes, such as increased screen time, automated transportation, and sedentary jobs, have led to reduced physical activity, especially among urban residents^28,29^. Age-specific analyses further revealed that older birth cohorts presented higher dietary risk-related mortality, possibly reflecting historical dietary patterns and nutritional transitions^30^. Similarly, males have shown an increasing trend of physical inactivity-attributable mortality in recent years, possibly because of the occupational transition from traditionally male-dominated manual labor to sedentary work patterns, driven by digitalization and remote working practices. In addition, males are usually regarded as primary breadwinners, which often leads to prioritizing career development over exercise and may have contributed to their declining physical activity levels. These findings highlight the need for targeted interventions that address both dietary risks and physical inactivity challenges.

### Strengths and limitations

Our study has several strengths. First, we utilized high-quality mortality data from the GBD database, enabling comprehensive temporal trend analyses over multiple decades. Second, our multidimensional analyses incorporating age, period, and cohort effects provided insights into both temporal trends and generational patterns. Additionally, the comparative analysis between Chinese and global trends offers a valuable context for understanding the unique challenges faced by China in young-onset stroke prevention.

However, our study was not without limitations. Although the GBD database is widely recognized for its reliability and comprehensive coverage^31^, our exclusive reliance on this single data source limited our ability to conduct external validation of the findings. The incorporation of alternative data sources should be utilized in the future to provide additional verification rather than only GBD estimates. Second, our analysis focused exclusively on four modifiable lifestyle-related risk factors, whereas early-onset stroke may be attributed to various other etiological factors (e.g. sleep duration^32^) that potentially contribute to disease burden. However, owing to data availability constraints, we were unable to include these additional risk factors in our analysis, and the potential interactive effects^33^ among those lifestyle factors on mortality were not considered. Third, our analysis did not adjust for potential confounders such as healthcare access differences and other contextual determinants that may influence the observed trends. Since the mortality and DALYs data were obtained directly from the GBD database, detailed quantitative information on these confounders was not available for China or global datasets. This residual confounding may have impacted our results and should be considered when the findings are interpreted. Fourth, our study used Joinpoint regression analysis, which assumes that the dependent variables follow a Poisson distribution and that identified trend changes represent true shifts in the underlying data. However, these assumptions may not fully capture complex population dynamics, and residual confounding may still exist. Furthermore, Joinpoint regression identifies associations over time but does not establish causality, and our findings should be interpreted as correlations rather than definitive causal relationships. Fifth, our macrolevel analysis of the contributions of these four lifestyle factors to early-onset stroke mortality among Chinese adults aged 25–54 years may mask regional heterogeneity. Owing to data limitations, regionspecific analyses were not possible, restricting the development of more targeted prevention strategies tailored to local cultural and behavioral patterns^34,35^. Additionally, since this study compares aggregated national (China) and global estimates without regional breakdowns, excluding regions with limited or poor-quality data for sensitivity analyses was not feasible. Although the GBD database uses advanced modeling to address data gaps, residual uncertainties remain. These limitations should be considered when the findings are interpreted. Taken together, these limitations highlight the need for future research incorporating multiple data sources, additional risk factors, region-specific analyses, and methodological approaches that further address confounding and causal factors to deepen the understanding of early-onset stroke determinants.

## CONCLUSIONS

Our findings highlight trends in lifestyle-related risk factors contributing to stroke burdens among Chinese adults aged 25–54 years, with notable disparities between mortality and disability patterns. While females presented patterns similar to global levels, males experienced significantly greater burdens in terms of both mortality and DALYs due to smoking, alcohol use, physical inactivity, and dietary risks. The greater disability burden, as indicated by steeper age effects in DALYs despite more moderate mortality trends, points to a substantial long-term disability burden. Public health strategies focusing on young and middle-aged adults are crucial for reducing both younger-onset stroke mortality and long-term disability. Special attention may be needed for young males, particularly with respect to increasing alcohol-related burdens, but population-wide early prevention efforts are essential for all groups. These findings provide evidence-based guidance for developing targeted yet inclusive prevention strategies to reduce the impact of early-onset stroke in China.

## Supplementary Material



## Data Availability

The data used for the analyses can be accessed openly through the GBD2021 online database (https://vizhub.healthdata.org/gbd-results/).
