# Supplementary material for: Lifestyle-attributable burden of young-onset stroke in Chinese and global populations aged 20–54 years: A three decades comparative study (1990–2021) using Global Burden of Disease study data
Source: Tob Induc Dis. 2025 Aug 29;23:10.18332/tid/208007. doi: 10.18332/tid/208007 (PMC12396192; doi:10.18332/tid/208007)
Supplement: Supplementary file 1 [file TID-23-122-s1.pdf]

Supplementary file Table 1. Absolute changes in mortality and DALYs rates of stroke due to tobacco, high alcohol use, dietary risks, and low physical activity among individuals aged 20–54 years in 1990 and 2021, with temporal trends over this period: secondary analysis of Global Burden of Disease (GBD) data.

			Tobacco			High alcohol use			Dietary risks			Low physical activity		
	Region	Sex	Value (1990)	Value (2021)	Value Change (95%CI)	Value (1990)	Value (2021)	Value Change (95%CI)	Value (1990)	Value (2021)	Value Change (95%CI)	Value (1990)	Value (2021)	Value Change (95%CI)
Death	China	Both	11.826	8.710	-3.116 (-6.387, 0.155)	1.970	2.148	0.178 (-2.450, 2.806)	8.230	4.737	-3.493 (-10.386, 3.400)	0.228	0.258	0.030 (-0.196, 0.256)
		Male	18.272	14.733	-3.539* (-6.232, -0.846)	3.560	4.006	0.446 (-1.310, 2.202)	9.941	6.793	-3.148 (-7.478, 1.182)	0.199	0.287	0.088 (-0.049, 0.225)
		Female	4.883	2.304	-2.579* (-3.522, -1.636)	0.256	0.171	-0.085 (-0.230, 0.060)	6.388	2.550	-3.838* (-6.842, -0.834)	0.260	0.227	-0.033 (-0.124, 0.058)
	Global	Both	7.569	4.688	-2.881* (-4.357, -1.405)	1.180	1.001	-0.179 (-0.717, 0.359)	5.487	3.318	-2.169* (-3.758, -0.580)	0.228	0.204	-0.024 (-0.076, 0.028)
		Male	11.333	7.591	-3.742* (-4.804, -2.680)	2.016	1.810	-0.206 (-0.920, 0.508)	6.578	4.301	-2.277* (-3.613, -0.941)	0.195	0.194	-0.001 (-0.061, 0.059)
		Female	3.709	1.733	-1.976* (-2.504, -1.448)	0.322	0.177	-0.145* (-0.281, -0.009)	4.368	2.317	-2.051* (-3.054, -1.048)	0.262	0.215	-0.047 (-0.120, 0.026)
DALYs	China	Both	558.562	421.469	-137.093 (-286.021, 11.835)	90.062	101.543	11.481 (-112.193, 135.155)	395.214	239.062	-156.152 (-473.906, 161.602)	13.380	16.711	3.331 (-9.391, 16.053)
		Male	853.124	701.261	-151.863 (-403.728, 100.002)	162.677	188.613	25.936 (-201.116, 252.988)	466.852	326.296	-140.556 (-556.101, 284.989)	10.341	15.674	5.333 (-9.639, 20.305)
		Female	241.277	123.909	-117.368* (-208.857, -25.879)	11.844	8.942	-2.902 (-17.330, 11.526)	318.049	146.288	-171.761 (-413.960, 70.438)	16.654	17.815	1.161 (-13.994, 16.316)
	Global	Both	365.975	230.454	-135.521* (-207.205, -63.837)	54.947	47.900	-7.047 (-73.555, 59.461)	273.460	172.455	-101.005 (-253.968, 51.958)	13.960	13.469	-0.491 (-9.703, 8.721)
		Male	538.855	366.088	-172.767* (-274.260, -71.274)	93.166	85.644	-7.522 (-121.117, 106.073)	318.784	214.390	-104.394 (-292.963, 84.175)	10.834	11.262	0.428 (-7.431, 8.287)
		Female	188.703	92.407	-96.296* (-148.908, -43.684)	15.758	9.484	-6.274 (-24.792, 12.244)	226.986	129.774	-97.212 (-223.615, 29.191)	17.166	15.715	-1.451 (-12.333, 9.431)

Note: "Value" indicates mortality rate for deaths, and absolute rate for DALYs; Data based on modeled estimates integrating multiple global sources; exact sample size not applicable.

* Indicates statistically significant changes in mortality rates at the α = 0.05 level.

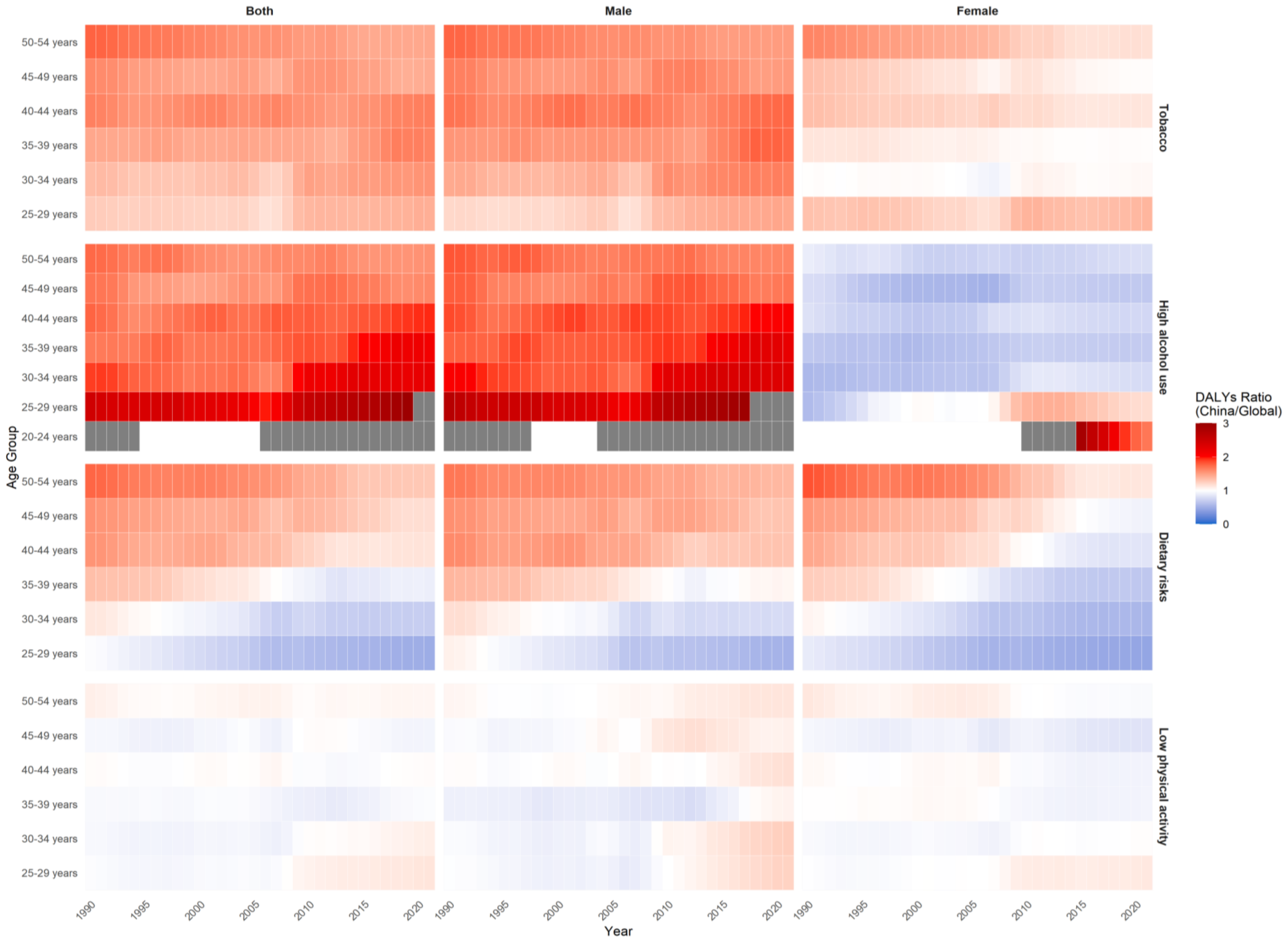

Supplementary file Figure 1. Ratio of risk factor-attributable DALY rates for stroke among individuals aged 25–54 years in China versus the global average, 1990–2021: secondary analysis of Global Burden of Disease (GBD) data.

Ratio = China's attributable mortality rate/global attributable mortality rate. Red (>1): China's rate higher than global average; White (=1): equal rate; Blue (<1): China's rate lower than global average; Grey: missing or abnormal data. Data based on modeled estimates integrating multiple global sources; exact sample size not applicable.

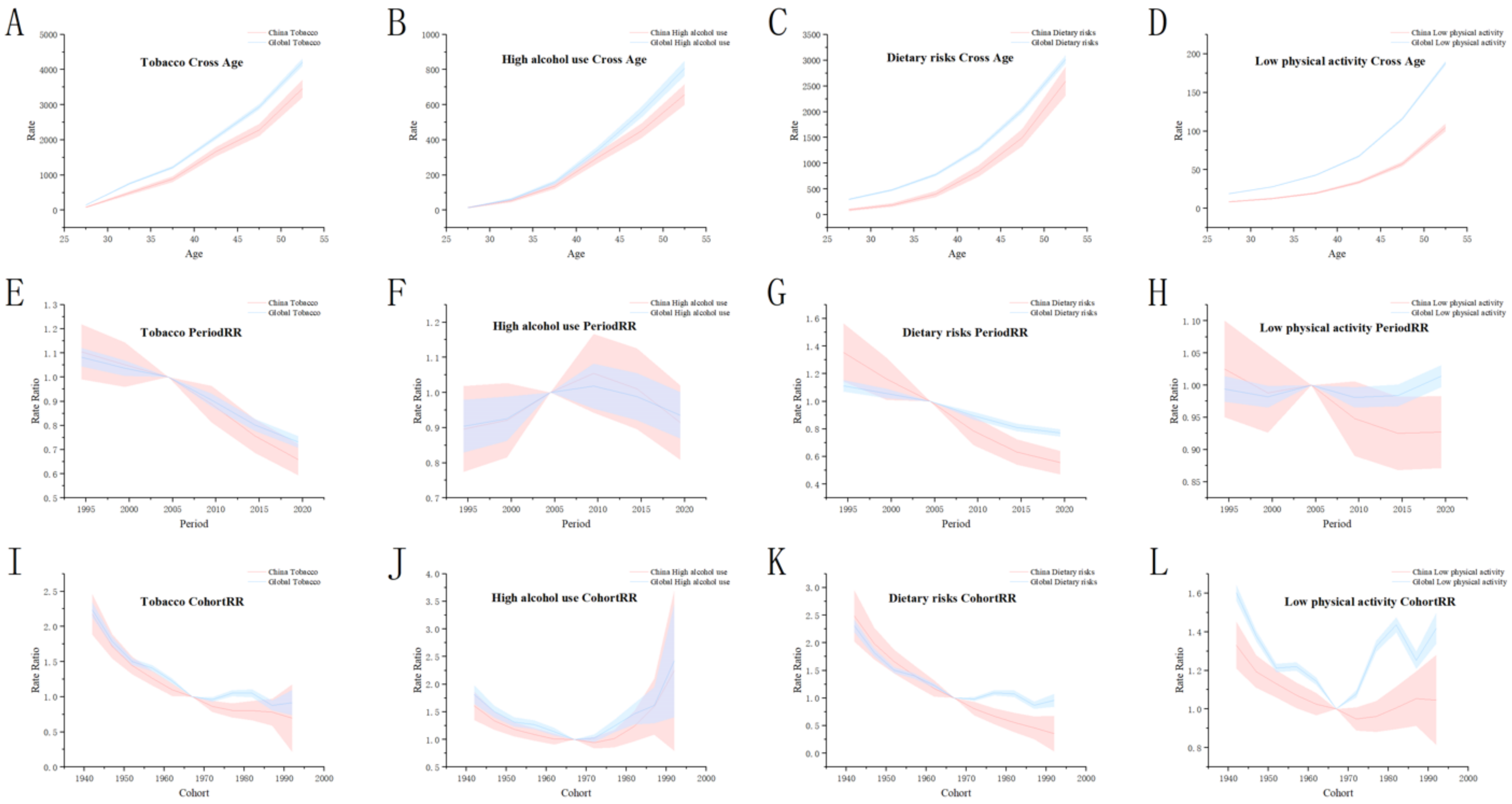

Supplementary file Figure 2. Comparison of age–period–cohort models for lifestyle factor–attributable stroke DALY rates among individuals aged 25–54 years in China and globally, 1992–2021: secondary analysis of Global Burden of Disease (GBD) data.

Panels (A-D) show age-specific trends attributable to (A) tobacco use, (B) high alcohol use, (C) dietary risks, and (D) low physical activity.

Panels (E-H) show period-specific trends attributable to (E) tobacco use, (F) high alcohol use, (G) dietary risks, and (H) low physical activity.

Panels (I-L) show cohort-specific trends attributable to (I) tobacco use, (J) high alcohol use, (K) dietary risks, and (L) low physical activity.

The red line represents China; the blue line represents the global average. Shaded areas denote 95% confidence intervals.

Data based on modeled estimates integrating multiple global sources; exact sample size not applicable.

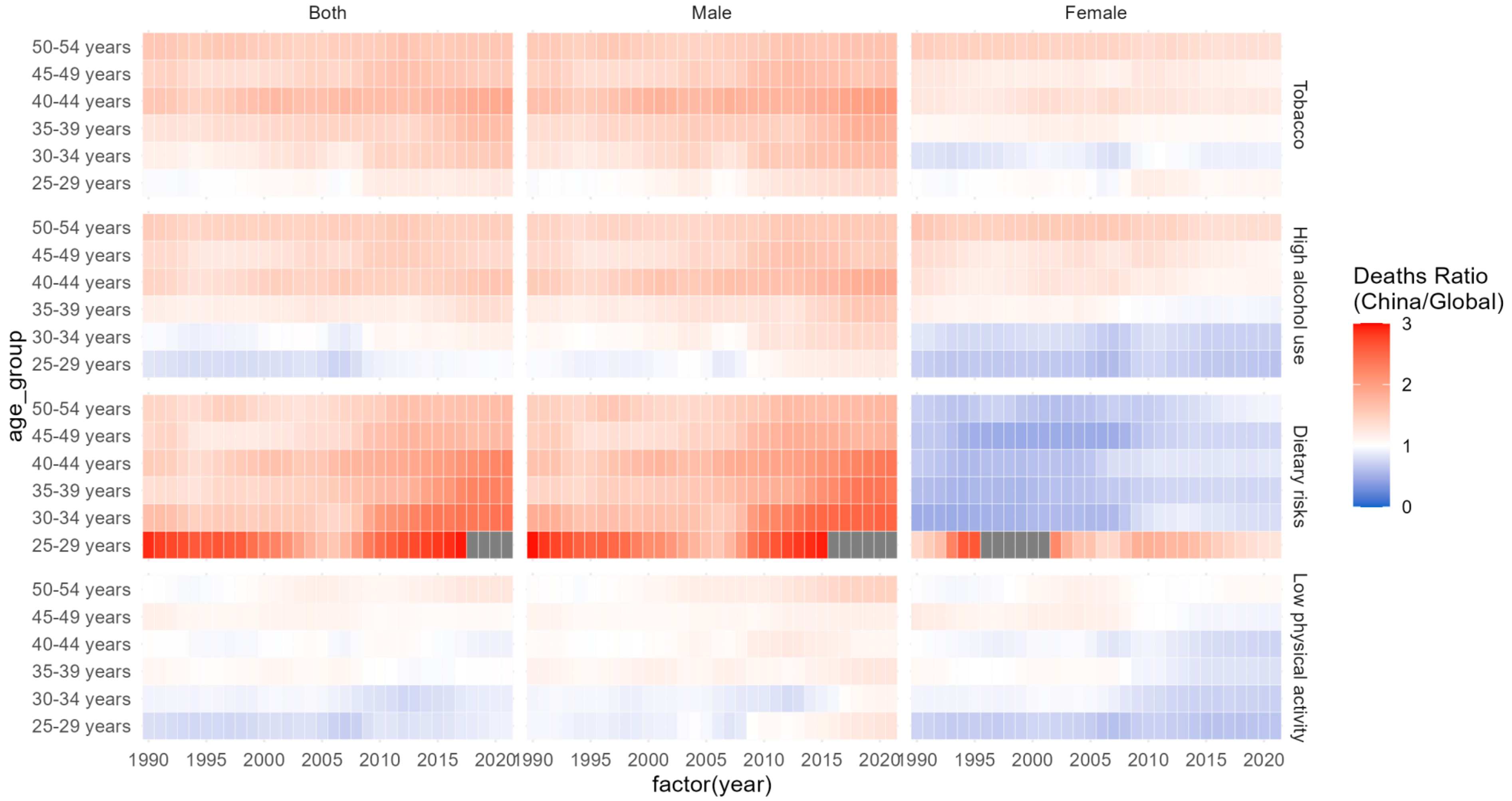

Supplementary file Figure 3. Risk factor–attributable mortality rate ratio for ischemic stroke among individuals aged 25–54 years in China versus the global average, 1990–2021: secondary analysis of Global Burden of Disease (GBD) data.

Ratio = China's attributable mortality rate/global attributable mortality rate. Red (>1): China's rate higher than global average; White (=1): equal rate; Blue (<1): China's rate lower than global average; Grey: missing or abnormal data. Data based on modeled estimates integrating multiple global sources; exact sample size not applicable.

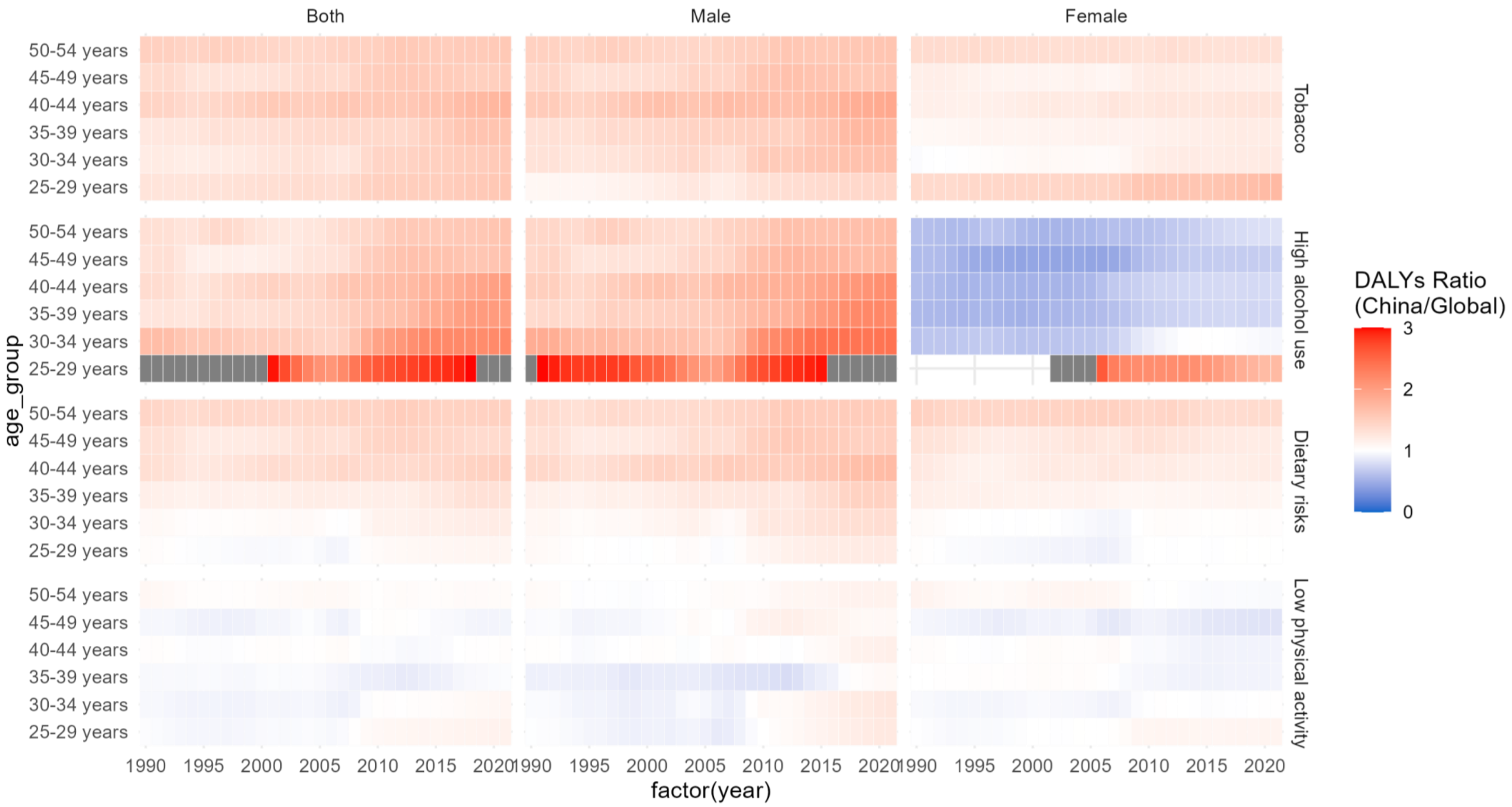

Supplementary file Figure 4. Ratio of risk factor-attributable DALY rates for ischemic stroke among individuals aged 25–54 years in China versus the global average, 1990–2021: secondary analysis of Global Burden of Disease (GBD) data.

Ratio = China's attributable mortality rate/global attributable mortality rate. Red (>1): China's rate higher than global average; White (=1): equal rate; Blue (<1): China's rate lower than global average; Grey: missing or abnormal data. Data based on modeled estimates integrating multiple global sources; exact sample size not applicable.

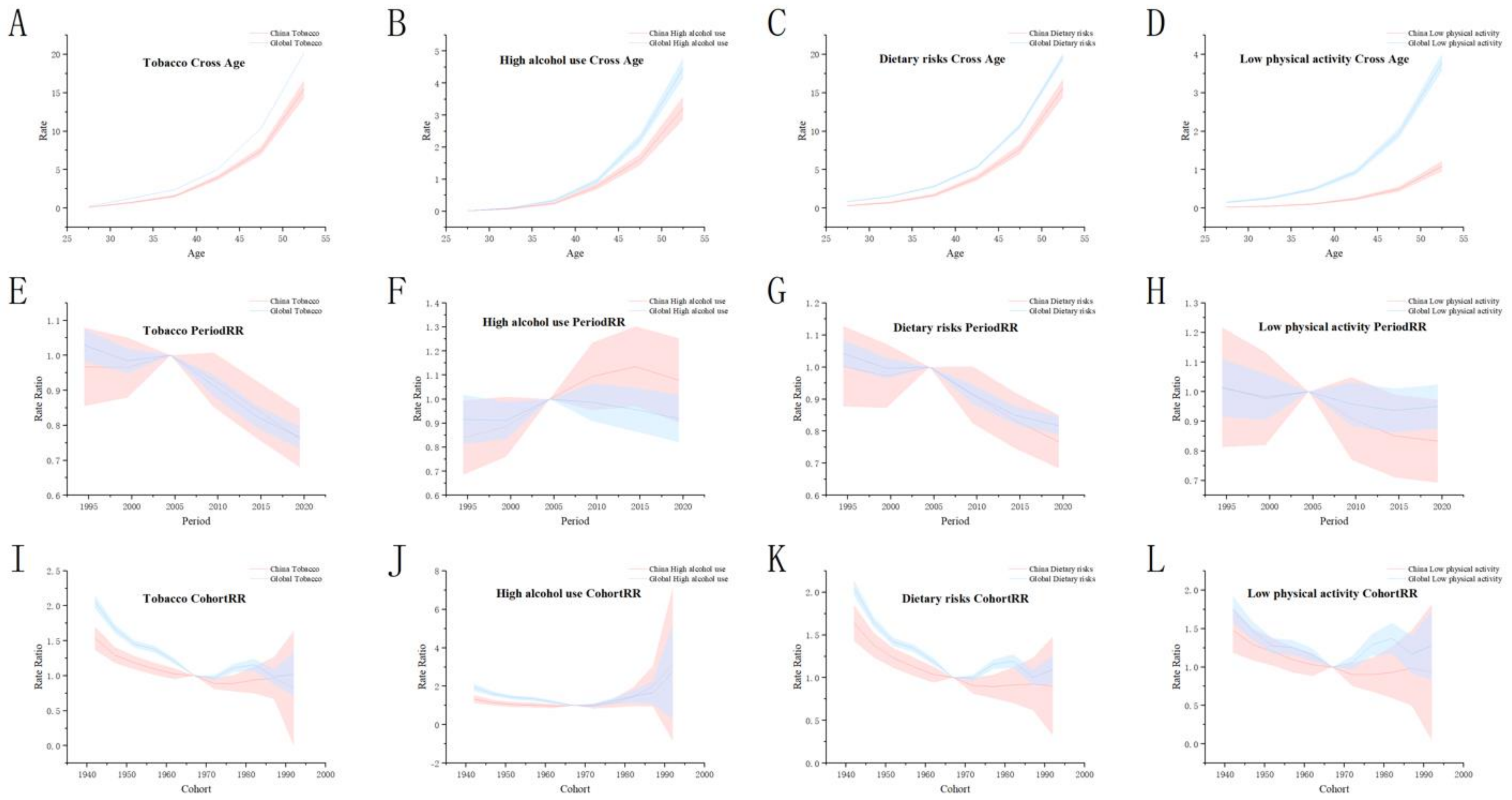

Supplementary file Figure 5. Comparison of age–period–cohort models for lifestyle factor–attributable ischemic stroke mortality rates among individuals aged 25–54 years in China and globally, 1992–2021: secondary analysis of Global Burden of Disease (GBD) data.

Panels (A-D) show age-specific trends attributable to (A) tobacco use, (B) high alcohol use, (C) dietary risks, and (D) low physical activity.

Panels (E-H) show period-specific trends attributable to (E) tobacco use, (F) high alcohol use, (G) dietary risks, and (H) low physical activity.

Panels (I-L) show cohort-specific trends attributable to (I) tobacco use, (J) high alcohol use, (K) dietary risks, and (L) low physical activity.

The red line represents China; the blue line represents the global average. Shaded areas denote 95% confidence intervals.

Data based on modeled estimates integrating multiple global sources; exact sample size not applicable.

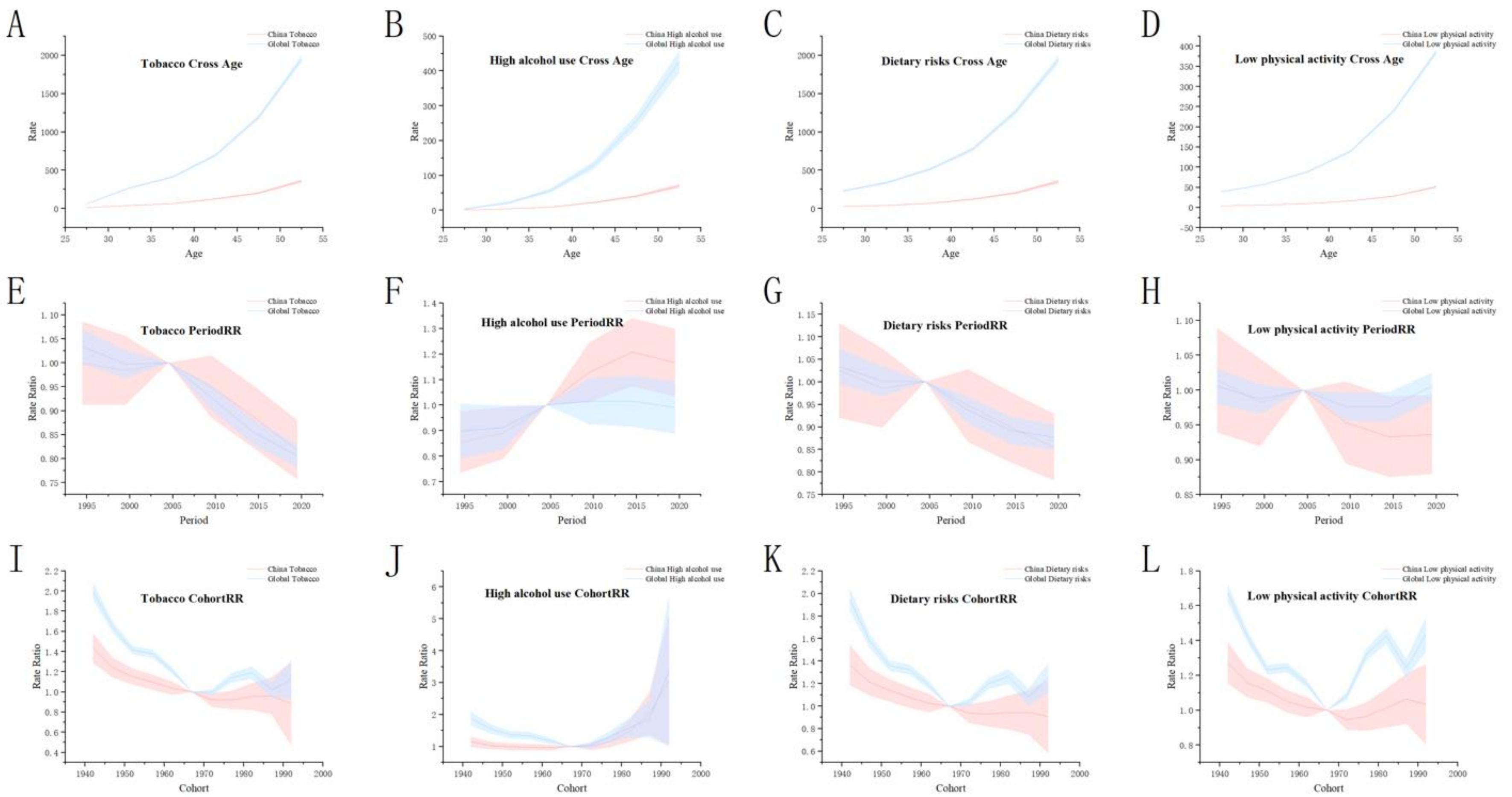

Supplementary file Figure 6. Comparison of age–period–cohort models for lifestyle factor–attributable ischemic stroke DALY rates among individuals aged 25–54 years in China and globally, 1992–2021: secondary analysis of Global Burden of Disease (GBD) data.

Panels (A-D) show age-specific trends attributable to (A) tobacco use, (B) high alcohol use, (C) dietary risks, and (D) low physical activity.

Panels (E-H) show period-specific trends attributable to (E) tobacco use, (F) high alcohol use, (G) dietary risks, and (H) low physical activity.

Panels (I-L) show cohort-specific trends attributable to (I) tobacco use, (J) high alcohol use, (K) dietary risks, and (L) low physical activity.

The red line represents China; the blue line represents the global average. Shaded areas denote 95% confidence intervals.

Data based on modeled estimates integrating multiple global sources; exact sample size not applicable.

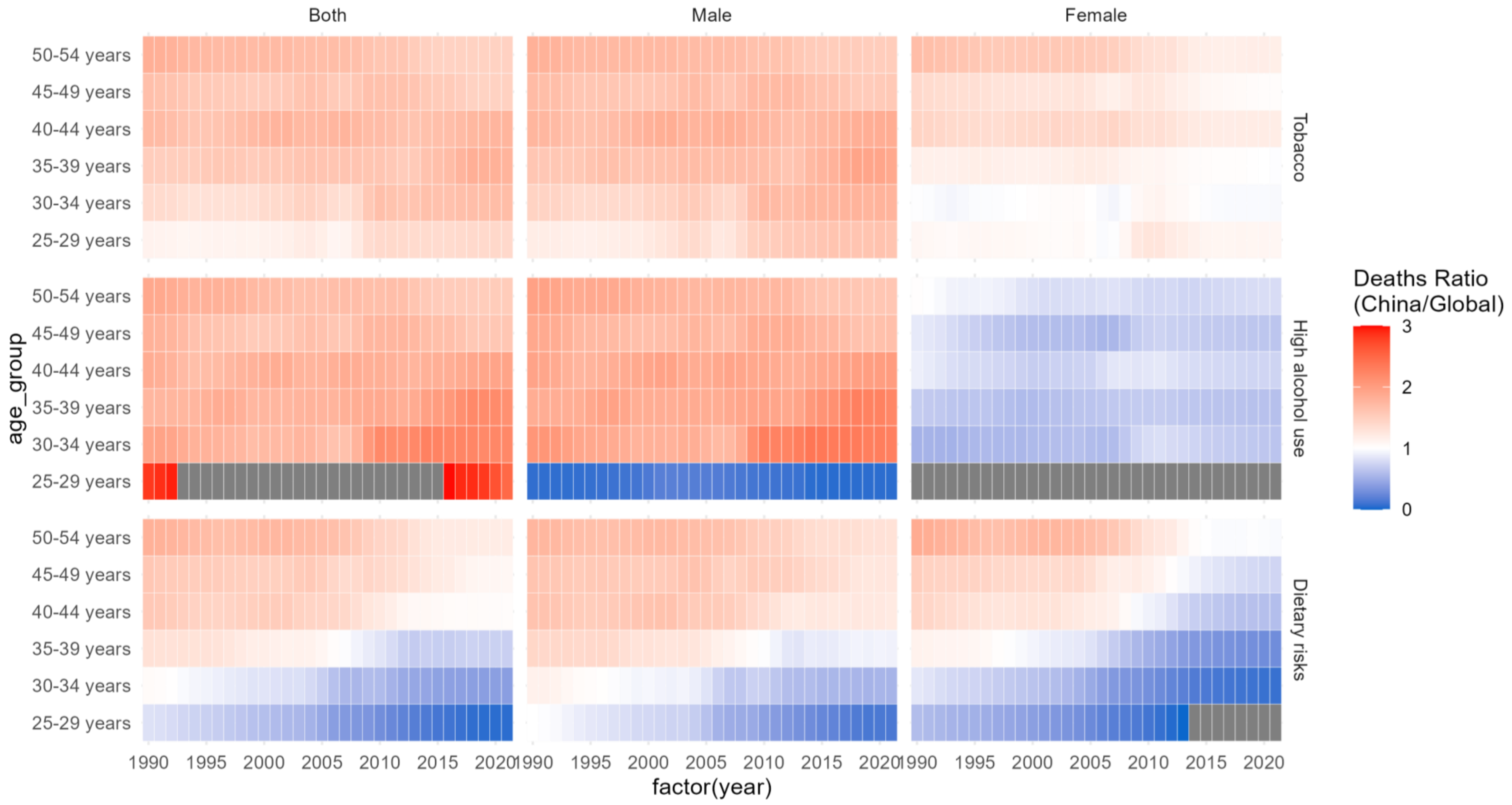

Supplementary file Figure 7. Risk factor–attributable mortality rate ratio for intracerebral hemorrhage among individuals aged 25–54 years in China versus the global average, 1990–2021: secondary analysis of Global Burden of Disease (GBD) data.

Ratio = China's attributable mortality rate/global attributable mortality rate. Red (>1): China's rate higher than global average; White (=1): equal rate; Blue (<1): China's rate lower than global average; Grey: missing or abnormal data. Data based on modeled estimates integrating multiple global sources; exact sample size not applicable.

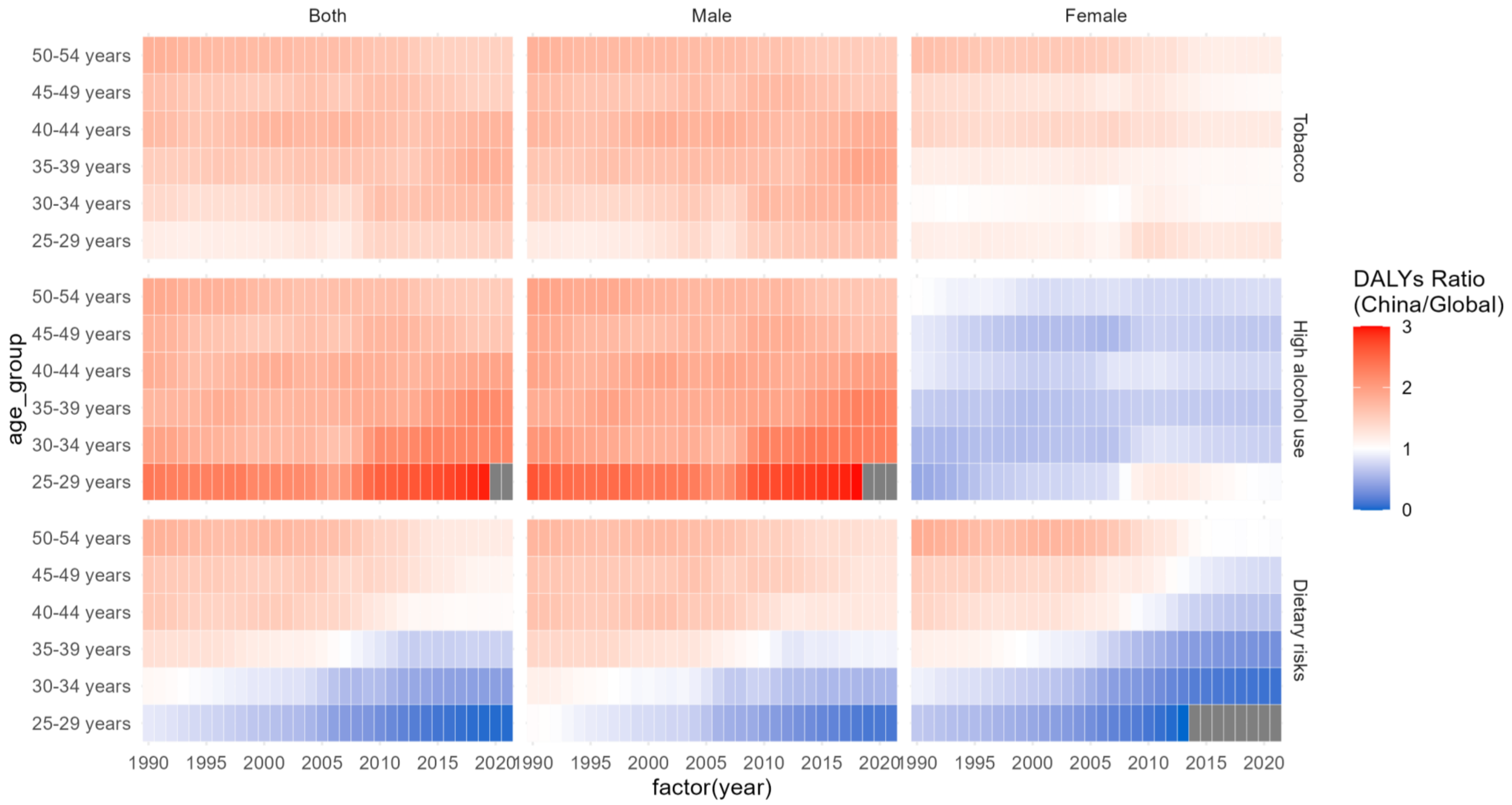

Supplementary file Figure 8. Ratio of risk factor–attributable DALY rates for intracerebral hemorrhage among individuals aged 25–54 years in China versus the global average, 1990–2021: secondary analysis of Global Burden of Disease (GBD) data.

Ratio = China's attributable mortality rate/global attributable mortality rate. Red (>1): China's rate higher than global average; White (=1): equal rate; Blue (<1): China's rate lower than global average; Grey: missing or abnormal data. Data based on modeled estimates integrating multiple global sources; exact sample size not applicable.

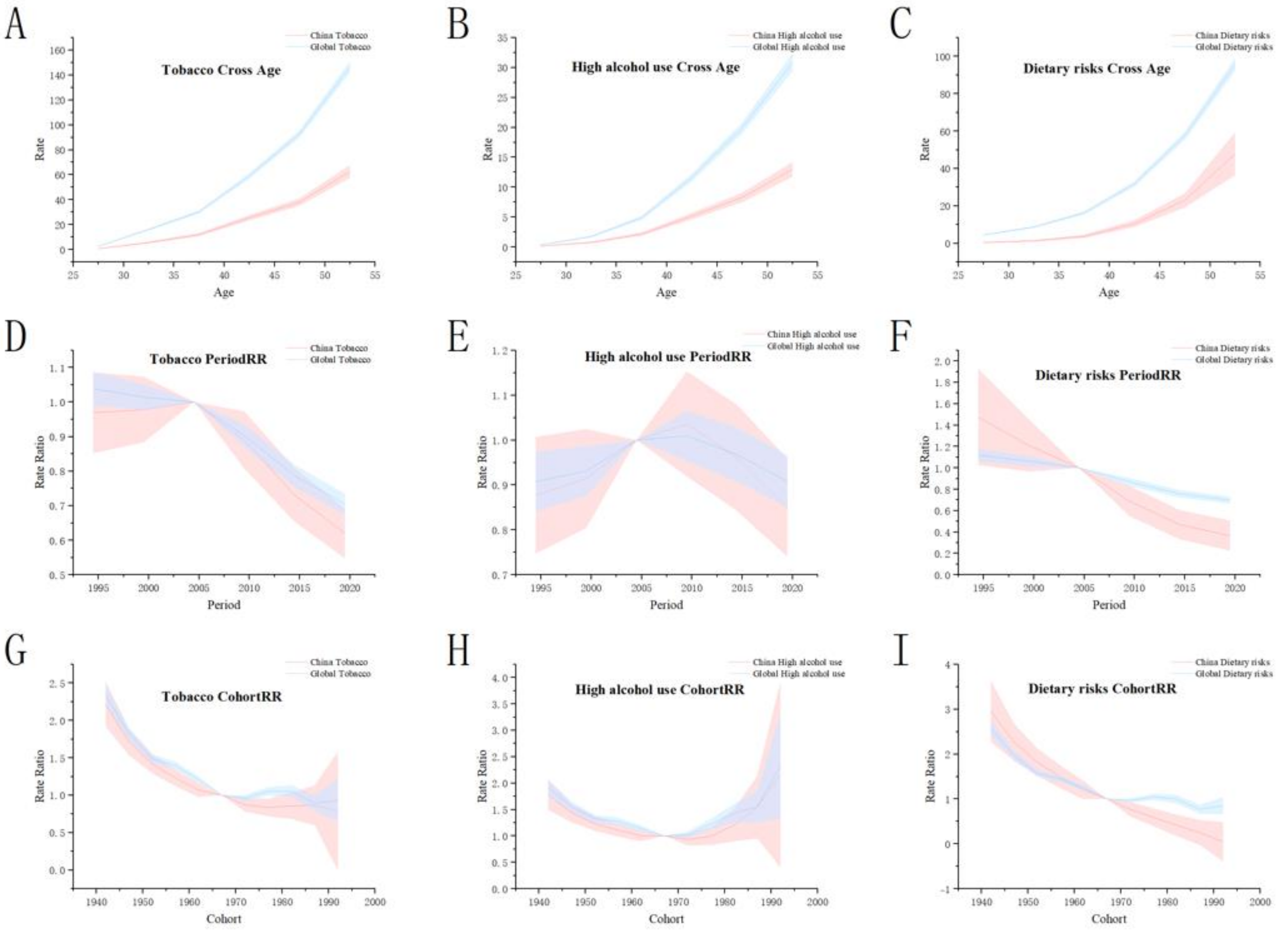

Supplementary file Figure 9. Comparison of age–period–cohort models for lifestyle factor–attributable intracerebral hemorrhage mortality rates among individuals aged 25–54 years in China and globally, 1992–2021: secondary analysis of Global Burden of Disease (GBD) data.

Panels (A-C) show age-specific trends attributable to (A) tobacco use, (B) high alcohol use, and (C) dietary risks.

Panels (D-F) show period-specific trends attributable to (D) tobacco use, (E) high alcohol use, and (F) dietary risks.

Panels (G-I) show cohort-specific trends attributable to (G) tobacco use, (H) high alcohol use, and (I) dietary risks.

The red line represents China; the blue line represents the global average. Shaded areas denote 95% confidence intervals.

Data based on modeled estimates integrating multiple global sources; exact sample size not applicable.

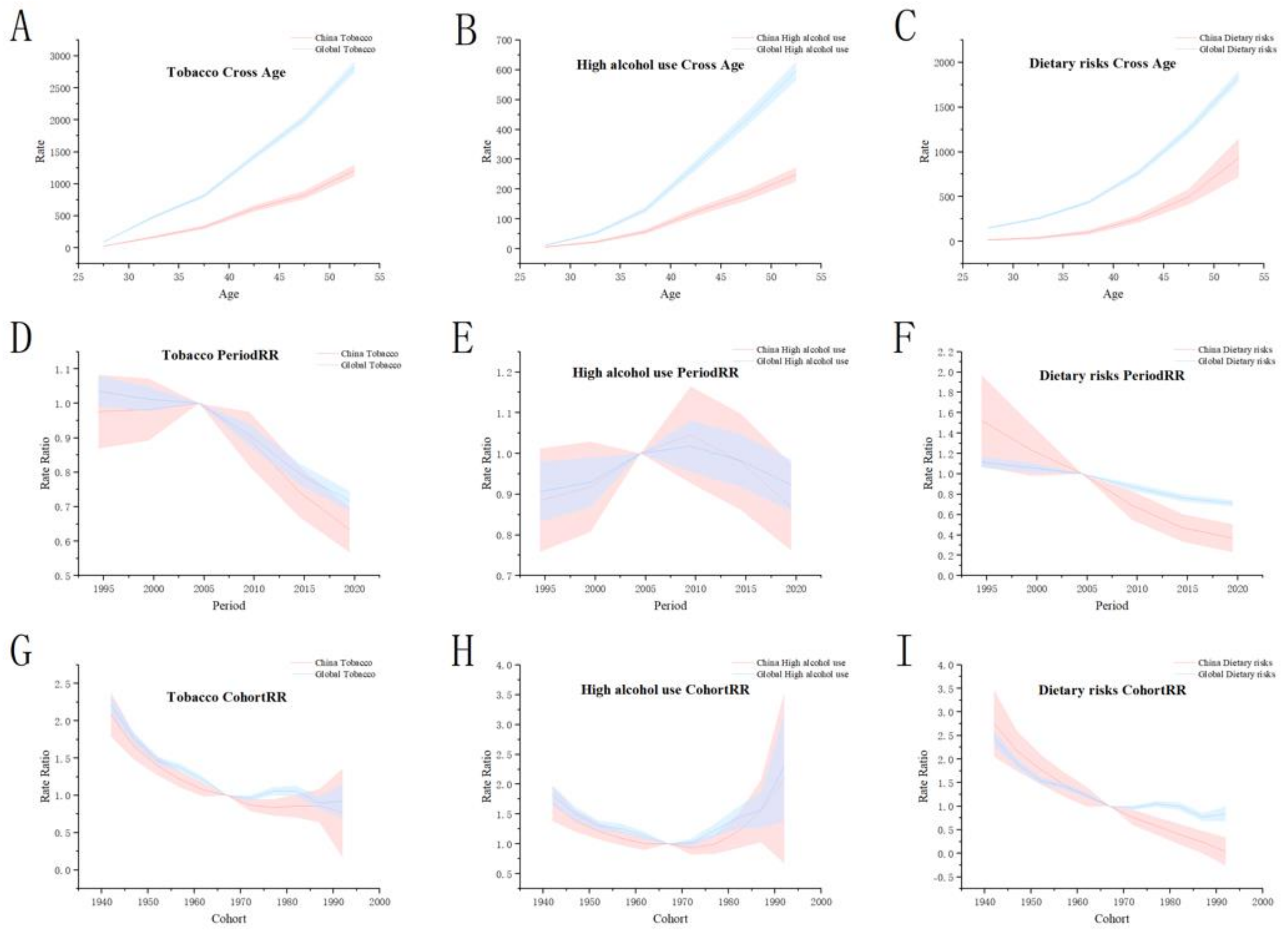

Supplementary file Figure 10. Comparison of age–period–cohort models for lifestyle factor–attributable intracerebral hemorrhage DALY rates among individuals aged 25–54 years in China and globally, 1992–2021: secondary analysis of Global Burden of Disease (GBD) data.

Panels (A-C) show age-specific trends attributable to (A) tobacco use, (B) high alcohol use, and (C) dietary risks.

Panels (D-F) show period-specific trends attributable to (D) tobacco use, (E) high alcohol use, and (F) dietary risks.

Panels (G-I) show cohort-specific trends attributable to (G) tobacco use, (H) high alcohol use, and (I) dietary risks.

The red line represents China; the blue line represents the global average. Shaded areas denote 95% confidence intervals.

Data based on modeled estimates integrating multiple global sources; exact sample size not applicable.

© 2025 Yang D. et al.
